# Supplementary figures and images for: Ecological Niche Differentiation and Distribution Dynamics Revealing Climate Change Responses in the Chinese Genus Dysosma
Source: Plants (Basel). 2026 Jan 5;15(1):162. doi: 10.3390/plants15010162 (PMC12788128; doi:10.3390/plants15010162)

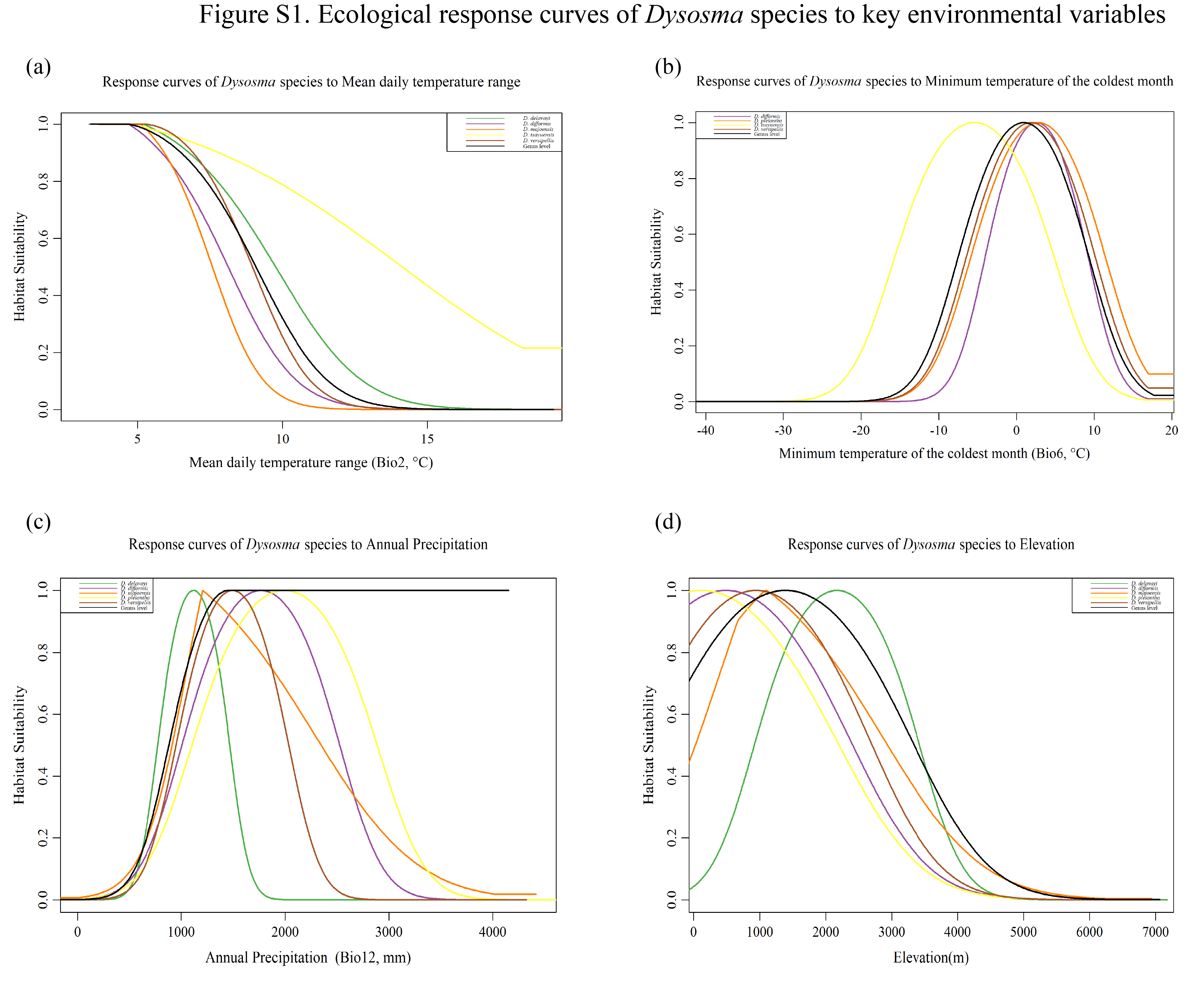

Supplement: Supplementary file 1 [file plants-15-00162-s001.zip › Figure S1. Ecological response curves of Dysosma species to key environmental variables.png]

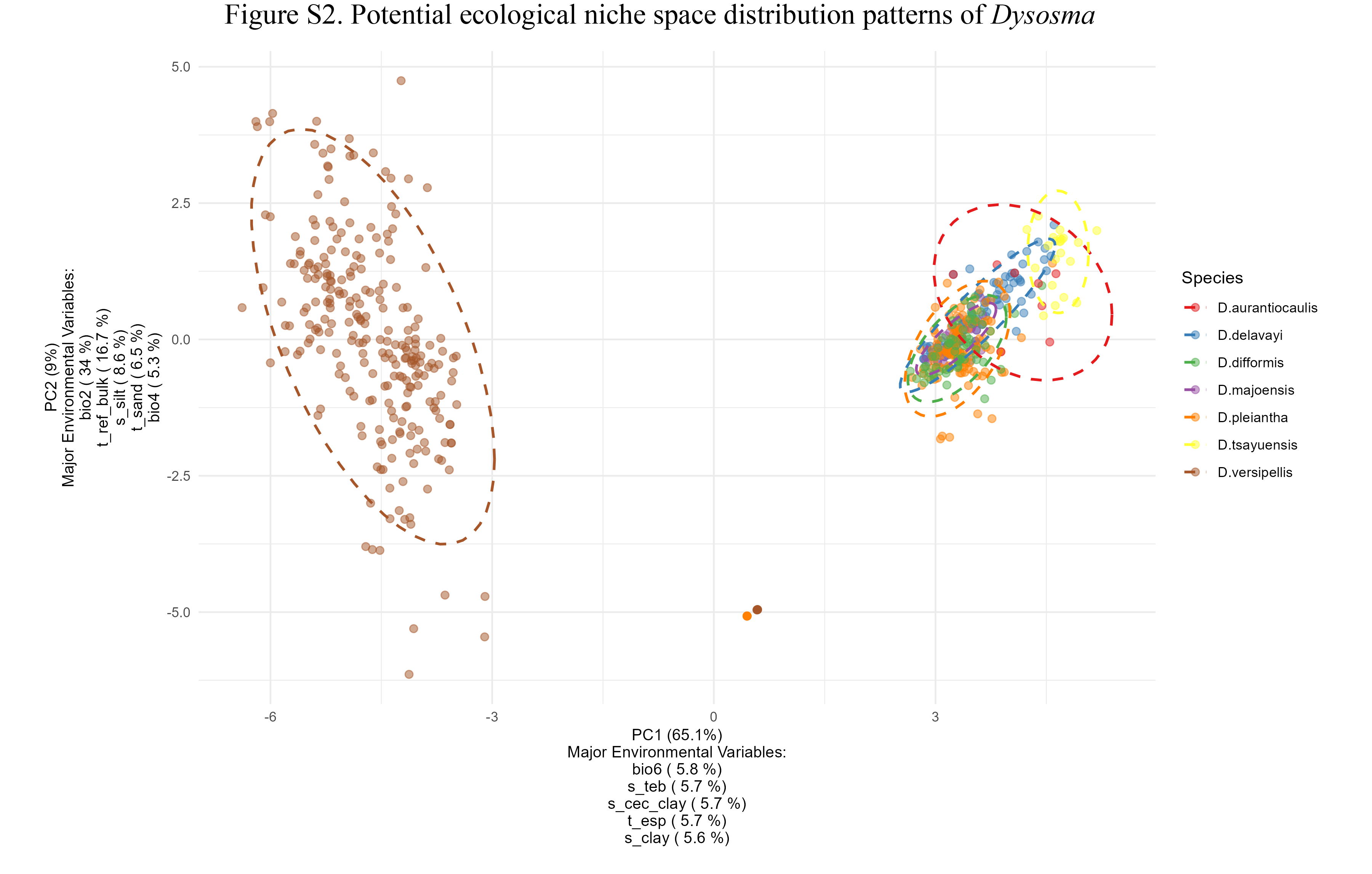

Supplement: Supplementary file 1 [file plants-15-00162-s001.zip › Figure S2. Potential ecological niche space distribution patterns of Dysosma.png]

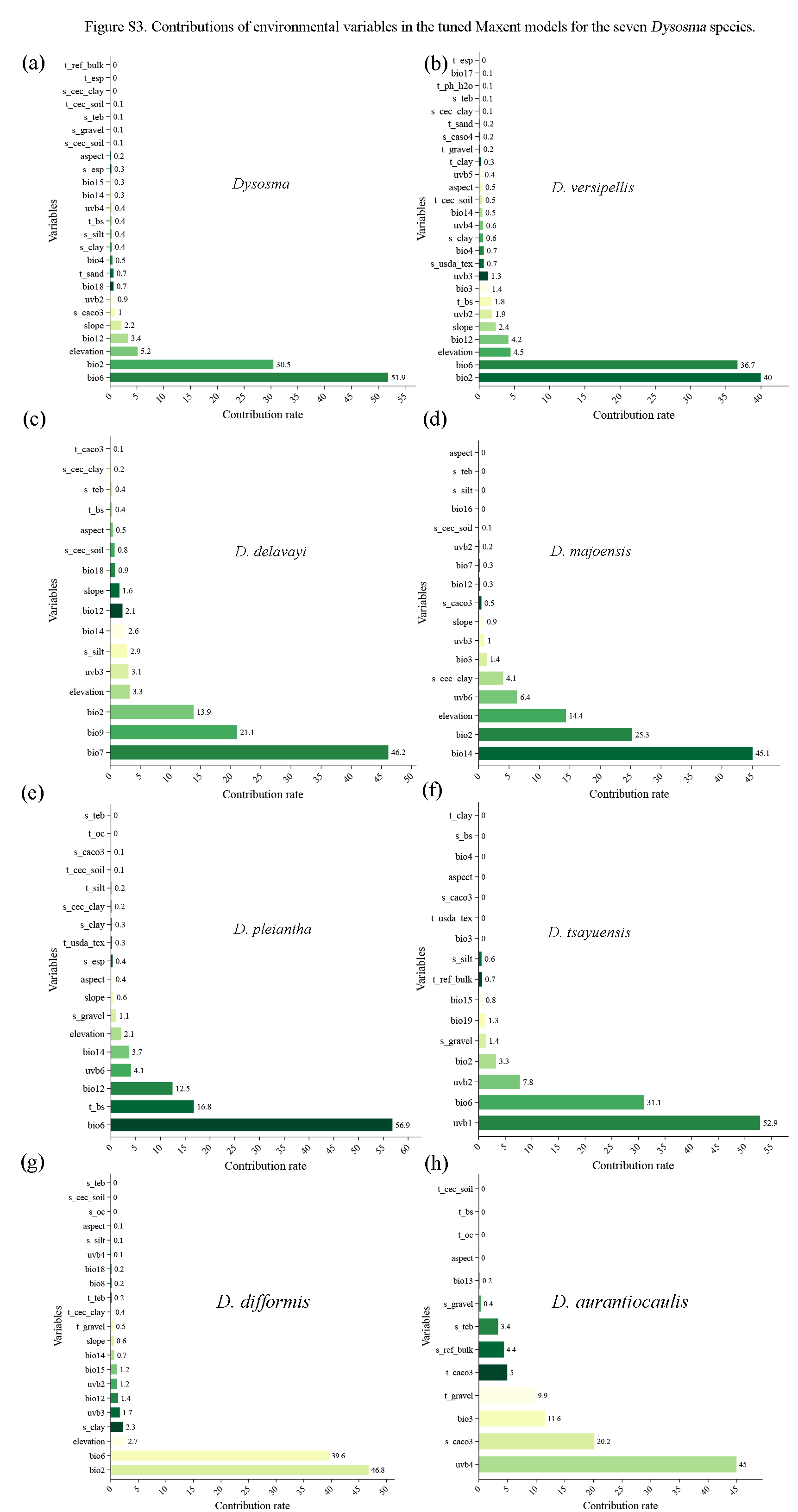

Supplement: Supplementary file 1 [file plants-15-00162-s001.zip › Figure S3. Contributions of environmental variables in the tuned Maxent models for the seven Dysosma species.png]

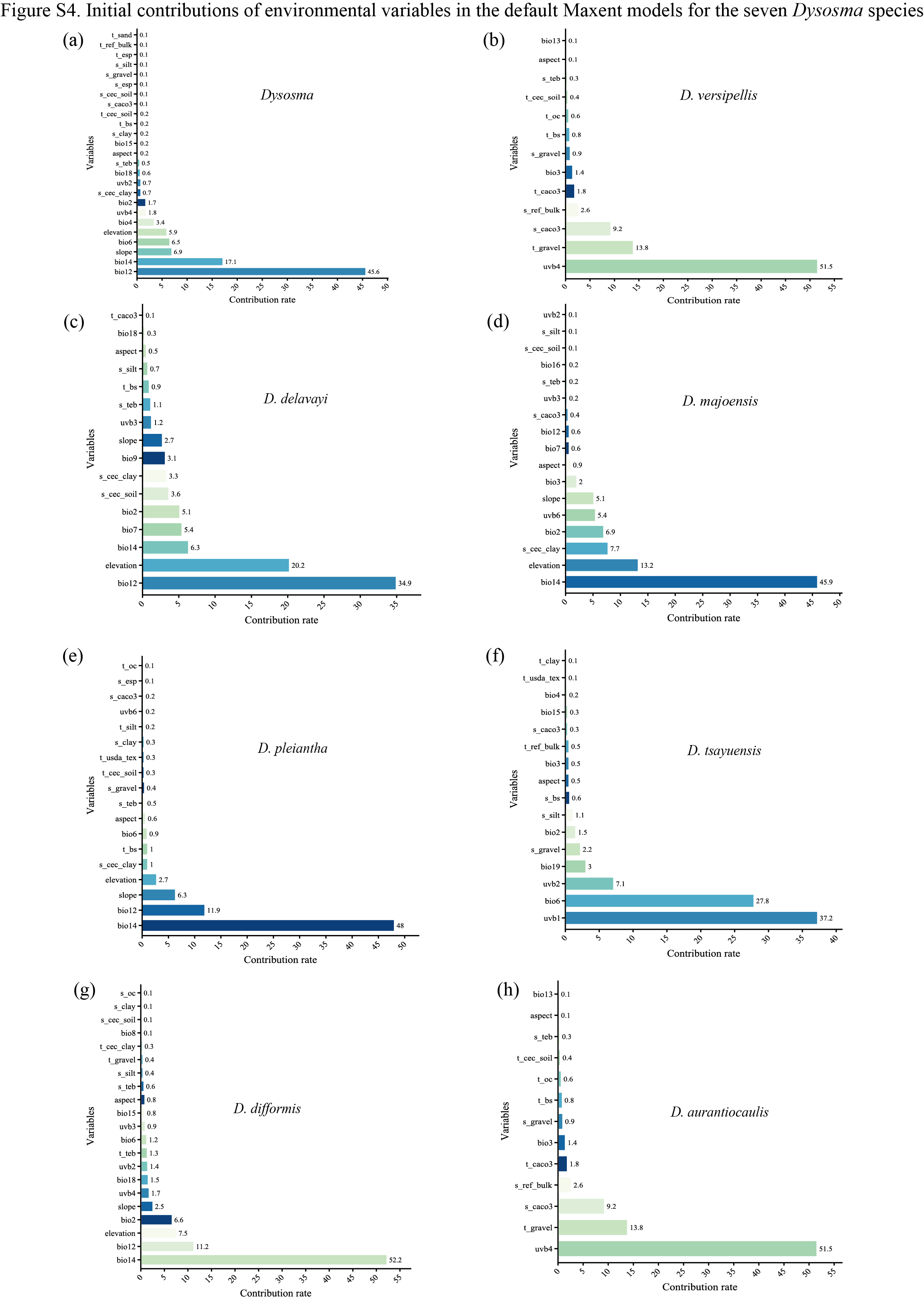

Supplement: Supplementary file 1 [file plants-15-00162-s001.zip › Figure S4. Initial contributions of environmental variables in the default Maxent models for the seven Dysosma species.png]

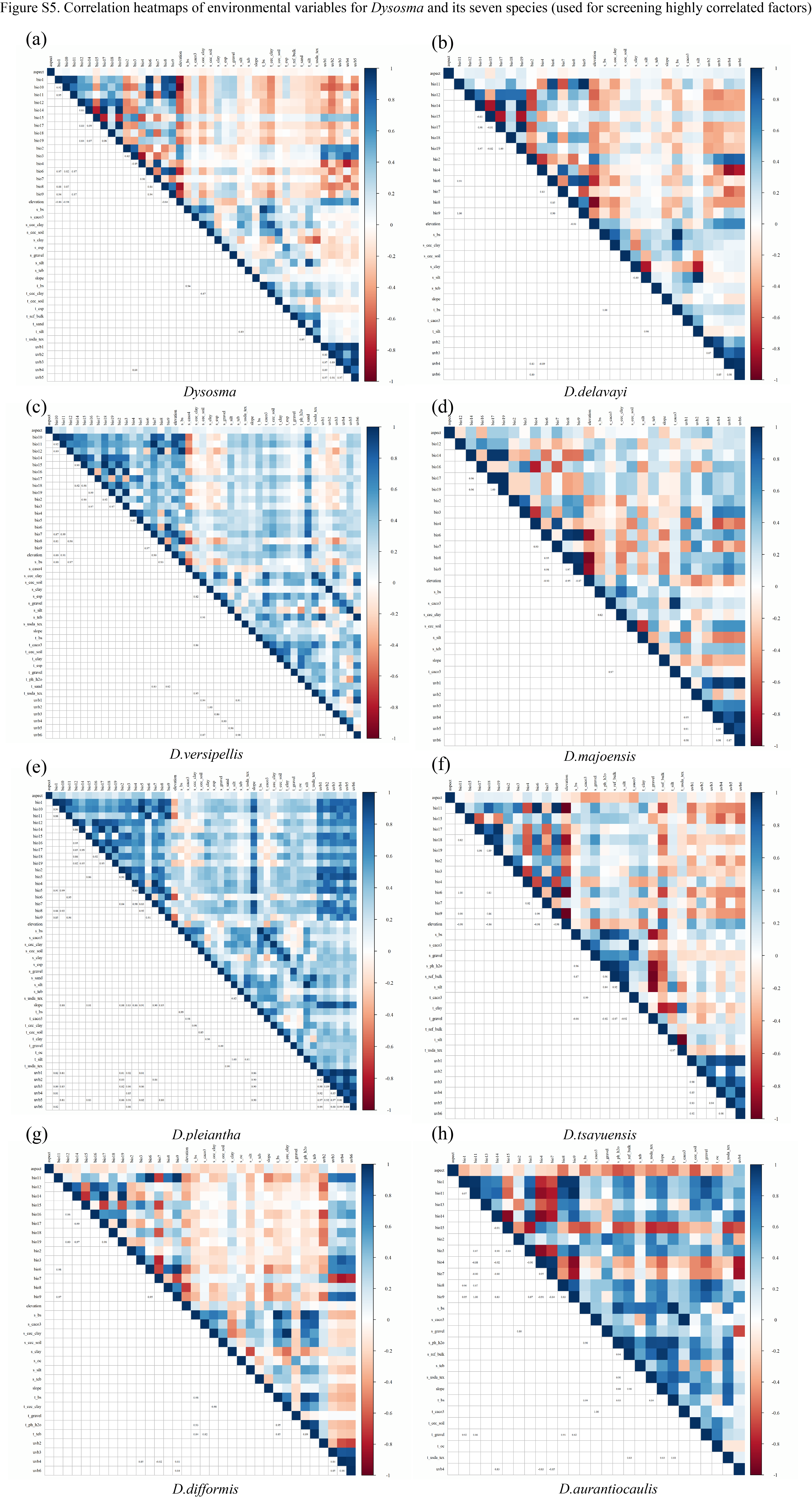

Supplement: Supplementary file 1 [file plants-15-00162-s001.zip › Figure S5. Correlation heatmaps of environmental variables for Dysosma and its seven species (used for screening highly correlated factors).png]
